# Supplementary material for: A phylogenomic resolution of the sea urchin tree of life
Source: BMC Evol Biol. 2018 Dec 13;18:189. doi: 10.1186/s12862-018-1300-4 (PMC6293586; doi:10.1186/s12862-018-1300-4)
Supplement: Supplementary file 1 — Figure S1. Visual representation of the occupancy of the matrix employed. Figure S2: Phylogenetic position of Arbacia punctulata and evidence for contamination. Figure S3. Residuals obtained from a linear regression of p-distances for each gene in the final alignment against its inferred orthologue in two other randomly selected taxa. Figure S4. Analyses excluding 345 outlier sequences detected by TreeShrink. (DOCX 5026 kb) [file 12862_2018_1300_MOESM1_ESM.docx]

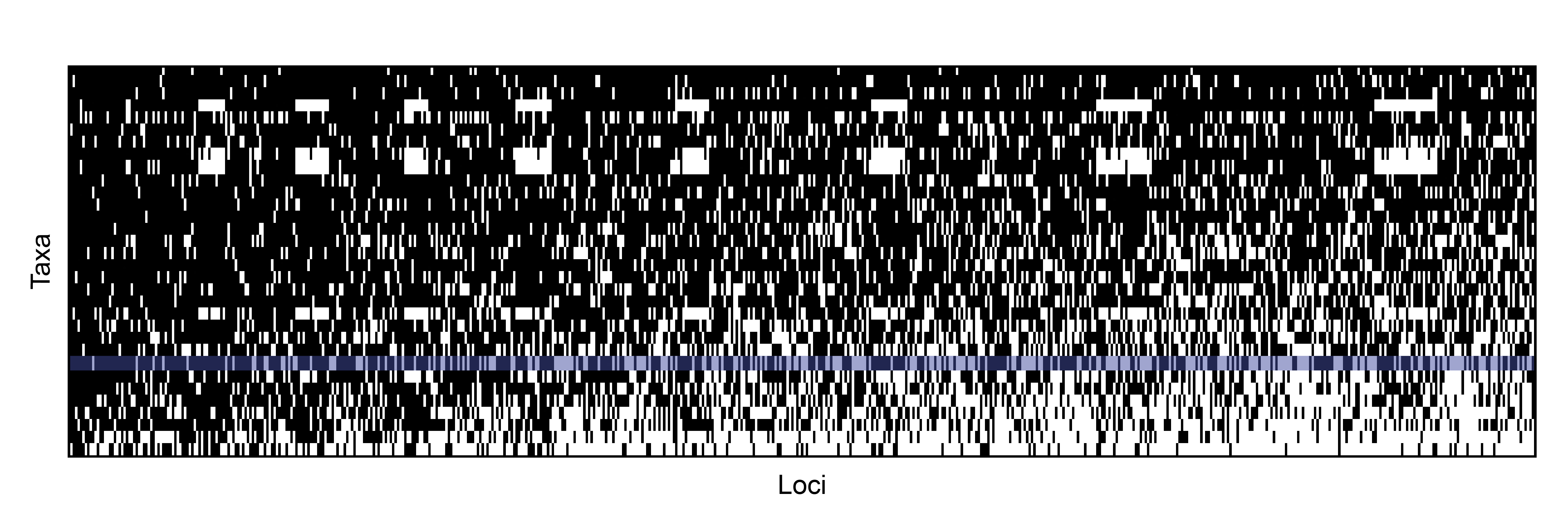


**Figure S1:** Visual representation of the occupancy of the matrix employed. Rows represent taxa and columns represent loci; presence is marked by a black cell (see also fig. 2A). The matrix is composed of 1,040 genes and 70% of cells are occupied. The excluded taxon, *Arbacia punctulata*, is highlighted in blue. The effective occupancy of this reduced matrix was 70.3%.


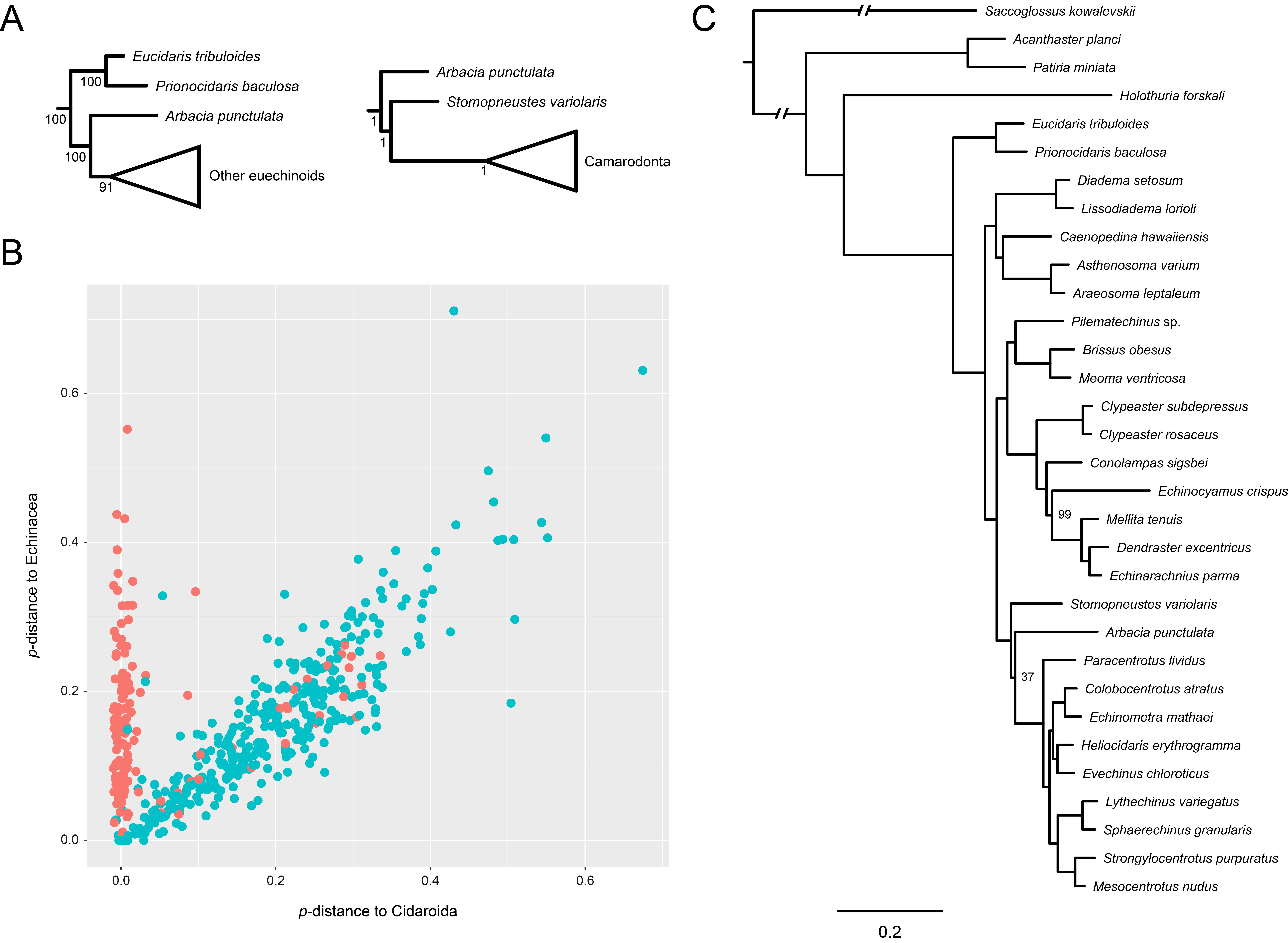


**Figure S2:** Phylogenetic position of *Arbacia punctulata* and evidence for contamination. **A.** Methods explored showed strong support for two different positions of *Arbacia* among echinoids. Both ML approaches, as well as ExaBayes, favored the topology on the left, with *Arbacia* sister to all other Euechinoidea. Support values are taken from the unpartitioned ML analysis. ASTRAL-II favored the more traditional position shown on the right, with *Arbacia* sister to a *Stomopneustes* + camarodont clade. PhyloBayes did not converge, with one chain sampling from each alternative topology. **B.** *P*-distances for all genes discovered in the transcriptome of *Arbacia* against a randomly selected echinacean and a randomly selected cidaroid. A subset of genes follows the expected linear trend of divergence with respect to these two groups, with slope < 1 consistent with its expected phylogenetic position within Echinacea (as in fig. A, right). A second group of genes has null distances to cidaroids, but non-zero distances to Echinacea (a small jitter has been added to the value of the x-axis). Inspection of gene trees reveals most of these show *Arbacia* sister to *Eucidaris tribuloides*, suggesting contamination might have occurred during handling of these two specimens. **C.** Once the putative contaminated sequences assigned to *Arbacia* are excluded from the alignment (i.e., all with sequences identical to those of cidaroids, of which we found 356 or 57.8% of the total recovered loci), its position within Echinacea is strongly supported (topology and support values correspond to a partitioned ML analysis; support is shown only if less than 100). The topology has *Arbacia* sister to Camarodonta, consistent with the position favored by morphological data, but the limited amount of data left precludes a robust resolution of echinacean relationships.


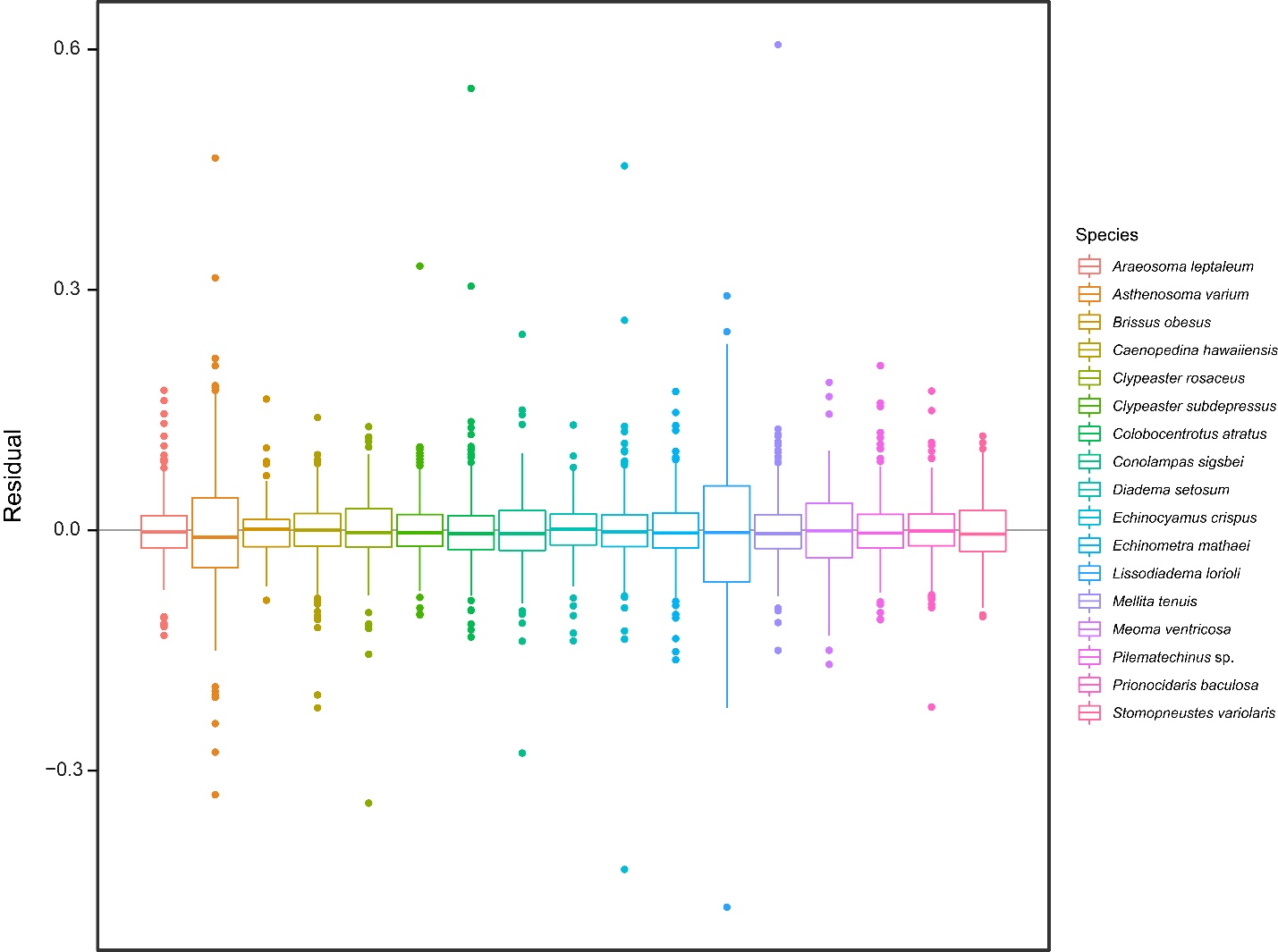


**Figure S3:** Residuals obtained from a linear regression of p-distances for each gene in the final alignment against its inferred orthologue in two other randomly selected taxa. Extreme outliers from the expected linear relationship represent a minor proportion of the total dataset (~ 1%), indicating cross-contamination is not expected to bias phylogenetic inference.


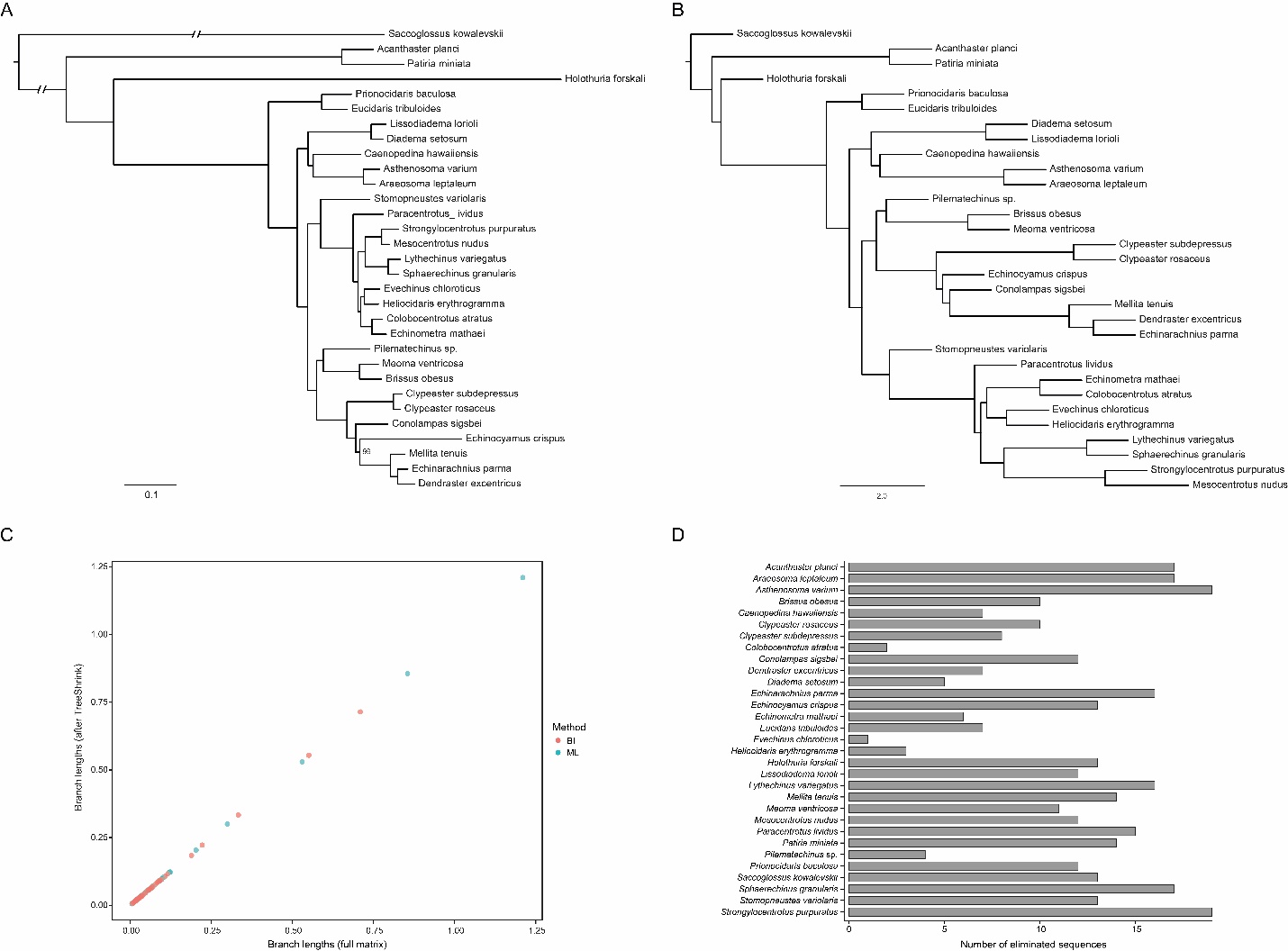


**Figure S4:** Analyses excluding 345 outlier sequences detected by TreeShrink. **A.** The topologies obtained under ML and BI approaches are identical to the ones obtained for the full dataset (Fig. 2A). Branch lengths depicted correspond to the ML tree. Support is maximum unless noted. **B.** The topology obtained using ASTRAL-II is identical to the one obtained for the full dataset (Fig. 3A). Support is maximum for all nodes. **C.** Branch lengths are not modified by the exclusion of outlier sequences (Pearson’s r = 0.99996, p < 10^-16^). **D.** Distribution of outlier sequences across terminals.
