# Supplementary material for: A phylogenomic resolution of the sea urchin tree of life
Source: BMC Evol Biol. 2018 Dec 13;18:189. doi: 10.1186/s12862-018-1300-4 (PMC6293586; doi:10.1186/s12862-018-1300-4)
Supplement: Supplementary file 2 — Table S1. Extraction, library preparation and sequencing protocols for all newly sequenced samples, as well as statistics output by Agalma for all transcriptomes included. (DOCX 18 kb) [file 12862_2018_1300_MOESM2_ESM.docx]

**Table S1:** Extraction, library preparation and sequencing protocols for all newly sequenced samples, as well as statistics output by Agalma for all transcriptomes included. The percentage of reads removed by Agalma includes ribosomal sequences, as well as adapter, quality and composition failures. Number of genes refers to the number of loci for a given terminal in the 70% occupancy matrix (1,040 loci; Fig. 2C). Number of genes for the included genomes: *Strongylocentrotus purpuratus* = 1,003; *Acanthaster planci* = 813; *Patiria miniata* = 736; *Saccoglossus kowalevskii* = 733.

| **Species** | **mRNA extraction** | **Library preparation** | **Illumina platform** | **Read pairs** | **Mean insert size** | **% removed by Agalma** | | | | **Reads pairs retained** | **Coding transcripts** | **Number of genes** |
| --- | --- | --- | --- | --- | --- | --- | --- | --- | --- | --- | --- | --- |
|  |  |  |  |  |  | **Ribosomal** | **Adapter** | **Quality** | **Composition** |  |  |  |
| *Araeosoma leptaleum* | Direct-zol RNA Miniprep Kit | KAPA Stranded RNA-Seq kit | HiSeq 4000 PE100 | 32,824,194 | 238.9 | 0.6 | 0.4 | 21.5 | 2.7 | 25,565,104 | 50,630 | 863 |
| *Arbacia punctulata* | - | - | - | 17,997,804 | 190.57 | 1.7 | 1.7 | 10.9 | 5.2 | 15,193,857 | 33,500 | 616 |
| *Asthenosoma varium* | Ambion PureLink RNA Mini Kit | Illumina TruSeq RNA kit | HiSeq 2500 PE125 | 67,577,575 | 191.4 | 0.05 | 0.09 | 18.8 | 0.4 | 58,110,236 | 59,996 | 948 |
| *Brissus obesus* | Direct-zol RNA Miniprep Kit | KAPA Stranded RNA-Seq kit | HiSeq 4000 PE100 | 30,348,945 | 237.1 | 1.3 | 2.2 | 23.4 | 3.2 | 22,564,575 | 19,655 | 511 |
| *Caenopedina hawaiiensis* | Direct-zol RNA Miniprep Kit | KAPA Stranded RNA-Seq kit | HiSeq 4000 PE100 | 38,105,472 | 259.4 | 1.8 | 0.2 | 27.4 | 1.0 | 27,462,085 | 29,231 | 796 |
| *Clypeaster rosaceus* | Ambion PureLink RNA Mini Kit | Illumina TruSeq RNA kit | HiSeq 2500 PE100 | 66,754,370 | 197.2 | 0.5 | 0.008 | 16.4 | 0.5 | 58,518,636 | 46,334 | 849 |
| *Clypeaster subdepressus* | Ambion PureLink RNA Mini Kit | Illumina TruSeq RNA kit | HiSeq 2500 PE100 | 84,561,739 | 203.4 | 0.7 | 0.01 | 15.2 | 0.6 | 74,475,117 | 59,277 | 910 |
| *Colobocentrotus atratus* | Direct-zol RNA Miniprep Kit | KAPA Stranded RNA-Seq kit | HiSeq 4000 PE100 | 42,546,671 | 300.7 | 10.3 | 0.03 | 15.0 | 1.3 | 31,932,004 | 31,539 | 742 |
| *Conolampas sigsbei* | Ambion PureLink RNA Mini Kit | Illumina TruSeq RNA kit | HiSeq 2500 PE100 | 41,056,403 | 198.5 | 0 | 0 | 16.4 | 0.2 | 36,373,405 | 52,059 | 749 |
| *Dendraster excentricus* | - | - | - | 28,537,534 | 197.8 | 0.6 | 1.4 | 9.4 | 2.7 | 25,365,590 | 15,346 | 312 |
| *Diadema setosum* | Direct-zol RNA Miniprep Kit | KAPA Stranded RNA-Seq kit | HiSeq 4000 PE100 | 33,702,734 | 262.4 | 0.6 | 0.7 | 26.9 | 3.8 | 24,047,902 | 10,940 | 245 |
| *Echinarachnius parma* | - | - | - | 40,256,046 | 212.0 | 0.2 | 0.007 | 17.5 | 0.6 | 34,984,323 | 44,563 | 825 |
| *Echinocyamus crispus* | Direct-zol RNA Miniprep Kit | KAPA Stranded RNA-Seq kit | HiSeq 4000 PE100 | 30,774,438 | 258.8 | 0.6 | 1.3 | 27.7 | 12.6 | 19,975,621 | 30,444 | 647 |
| *Echinometra mathaei* | Direct-zol RNA Miniprep Kit | KAPA Stranded RNA-Seq kit | HiSeq 4000 PE100 | 44,005,972 | 264.0 | 1.2 | 0.08 | 13.9 | 2.1 | 37,187,175 | 26,493 | 695 |
| *Eucidaris tribuloides* | - | - | - | 18,165,055 | 199.4 | 2.6 | 1.6 | 10.2 | 7.7 | 14,992,321 | 21,524 | 467 |
| *Evechinus chloroticus* | - | - | - | 23,380,500 | 164.2 | 4.3 | 0.1 | 17.1 | 0.7 | 19,367,504 | 32,768 | 748 |
| *Heliocidaris erythrogramma* | - | - | - | 34,293,765 | 169.9 | 0.2 | 0.002 | 12.6 | 3.8 | 29,951,034 | 18,662 | 879 |
| *Holothuria forskali* | - | - | - | 80,911,707 | 170.7 | 0.04 | 2.9 | 16.2 | 0.5 | 70,490,724 | 35,688 | 660 |
| *Lissodiadema lorioli* | Direct-zol RNA Miniprep Kit | KAPA Stranded RNA-Seq kit | HiSeq 4000 PE100 | 36,578,442 | 315.3 | 2.9 | 0.03 | 20.3 | 0.7 | 28,436,006 | 23,949 | 632 |
| *Lytechinus variegatus* | - | - | - | 30,644,263 | 274.8 | 0.4 | 0 | 15.6 | 0.8 | 26,812,944 | 34,772 | 822 |
| *Mellita tenuis* | Direct-zol RNA Miniprep Kit | KAPA Stranded RNA-Seq kit | HiSeq 4000 PE100 | 34,088,387 | 265.7 | 22.6 | 0.1 | 23.9 | 17.5 | 15,785,900 | 30,085 | 686 |
| *Meoma ventricosa* | Ambion PureLink RNA Mini Kit | Illumina TruSeq RNA kit | HiSeq 2500 PE100 | 46,897,779 | 184.0 | 0.1 | 0.03 | 13.7 | 0.6 | 42,125,813 | 29,761 | 891 |
| *Mesocentrotus nudus* | - | - | - | 28,823,832 | 256.6 | 1.1 | 0 | 8.2 | 0.7 | 26,207,098 | 42,007 | 901 |
| *Paracentrotus lividus* | - | - | - | 20,175,205 | 193.7 | 0.2 | 0.008 | 13.0 | 1.3 | 18,059,227 | 41,696 | 768 |
| *Pilematechinus* sp. | Direct-zol RNA Miniprep Kit | KAPA Stranded RNA-Seq kit | HiSeq 4000 PE100 | 34,233,585 | 275.9 | 2.8 | 0.09 | 24.1 | 2.2 | 25,134,883 | 25,442 | 550 |
| *Prionocidaris baculosa* | Direct-zol RNA Miniprep Kit | KAPA Stranded RNA-Seq kit | HiSeq 4000 PE100 | 38,619,001 | 269.9 | 0.4 | 0.1 | 13.2 | 3.5 | 32,839,869 | 27,647 | 616 |
| *Sphaerechinus granularis* | - | - | - | 41,722,413 | 181.9 | 0.1 | 0.004 | 21.1 | 0.5 | 35,404,566 | 52,806 | 862 |
| *Stomopneustes variolaris* | Direct-zol RNA Miniprep Kit | KAPA Stranded RNA-Seq kit | HiSeq 4000 PE100 | 34,056,015 | 257.1 | 0.4 | 0.5 | 25.5 | 5.5 | 24,486,356 | 28,587 | 821 |
